# The mycorrhiza-dependent defensin MtDefMd1 of *Medicago truncatula* acts during the late restructuring stages of arbuscule-containing cells

**DOI:** 10.1371/journal.pone.0191841

**Published:** 2018-01-25

**Authors:** Marian Uhe, Claudia Hogekamp, Rico M. Hartmann, Natalija Hohnjec, Helge Küster

**Affiliations:** Unit IV-Plant Genomics, Institute of Plant Genetics, Leibniz Universität Hannover, Hannover, Germany; Estacion Experimental del Zaidin, SPAIN

## Abstract

Different symbiotic and pathogenic plant-microbe interactions involve the production of cysteine-rich antimicrobial defensins. In *Medicago truncatula*, the expression of four *MtDefMd* genes, encoding arbuscular mycorrhiza-dependent defensins containing an N-terminal signal peptide and exhibiting some differences to non-symbiotic defensins, raised over the time of fungal colonization. Whereas the *MtDefMd1* and *MtDefMd2* promoters were inactive in cells containing young arbuscules, cells with fully developed arbuscules displayed different levels of promoter activities, indicating an up-regulation towards later stages of arbuscule formation. *MtDefMd1* and *MtDefMd2* expression was absent or strongly down-regulated in mycorrhized *ram1-1* and *pt4-2* mutants, known for defects in arbuscule branching or premature arbuscule degeneration, respectively. A ~97% knock-down of *MtDefMd1/MtDefMd2* expression did not significantly affect arbuscule size. Although overexpression of *MtDefMd1* in arbuscule-containing cells led to an up-regulation of *MtRam1*, encoding a key transcriptional regulator of arbuscule formation, no morphological changes were evident. Co-localization of an MtDefMd1-mGFP6 fusion with additional, subcellular markers revealed that this defensin is associated with arbuscules in later stages of their life-cycle. MtDefMd1-mGFP6 was detected in cells with older arbuscules about to collapse, and ultimately in vacuolar compartments. Comparisons with mycorrhized roots expressing a tonoplast marker indicated that MtDefMd1 acts during late restructuring processes of arbuscule-containing cells, upon their transition into a post-symbiotic state.

## Introduction

The vast majority of terrestrial plants is able to form arbuscular mycorrhizal (AM) symbioses with a group of obligate biotrophic fungi designated *Glomeromycota* [1, 2]. In return for the supply with hexoses [3] and presumably lipids in the form of palmitic acid [4], AM-fungal mycelia recruit nutrients, especially phosphate, from soils and transport them to the host [5]. It is thought that 450 million years ago, AM fungi facilitated the colonization of land by early plant species [6, 7].

Prior to a colonization of plant roots with AM fungi, strigolactones from the host trigger germination of fungal spores and growth of extraradical hyphae [8, 9]. The hyphae penetrate root epidermal cells via hyphopodia and spread in the cortex from this initial infection site [10, 11]. Both in the epidermis and in the subsequently colonized cells of the root, a pre-penetration apparatus, derived from structures of the cytoskeleton, precedes fungal entry [12]. In cells of the inner cortex, the fungus establishes arbuscules, highly branched, dendritic hyphal ends, being of particular importance for the exchange of nutrients [13]. Nutrient transfer between both partners involves a tight control of the microsymbiont's life-cycle by the host. Fungal arbuscules are regularly degraded after several days, and previously infected cells are restructured for subsequent re-infections [10, 14, 15].

Two AM-specific transcription factors act as key control switches of arbuscule development. With respect to arbuscule build-up, RAM1, together with other GRAS-transcription factors, controls proper arbuscule branching [16]. During the late stages of arbuscule development, the Myb transcription factor MYB1 activates a transcriptional program associated with arbuscule degeneration, leading to the production of different hydrolases and other arbuscule-degrading enzymes [17].

During the formation of active arbuscules, a suite of mycorrhiza-specific phosphate transporter- and other nutrient transporter genes is activated [18, 19]. The transfer of nutrients between fungi and plants throughout the AM symbiosis is mediated by the periarbuscular membrane (PAM), which permanently separates micro- and macrosymbionts [20]. Although the picture of PAM biogenesis, including polarized secretion for spatial expansion of the plasma membrane, is up to now not complete, some key players such as vesicle-associated membrane proteins (VAMPs), syntaxins, vapyrin, and EXO70i were identified [21, 22, 23, 24].

On the other hand, the process of PAM degradation is less clear, and the switch to cell-autonomous arbuscule degeneration is not yet defined, although important components of the degradation program were identified [17]. In this context, Kobae *et al*. [14] raised the question, why infection units composed of arbuscules in different stages of their life-cycle dissappear in a synchronized manner.

Many eukaryotic cells express genes encoding bioactive, channel-forming amphipathic peptides, such as those belonging to the defensin superfamily [25]. Defensins are not legume-specific, and several members of the defensin family have long been known for their role as defense-related peptides in plants [26]. It was proven that some defensins are translated during the defence reaction towards fungal pathogens [27], while filamentous fungi as well as yeast are considered to be targets as well. Characteristic for plant defensin proteins are an α-helix and a three-stranded, anti-parallel β-sheet, being interconnected by four disulfide bonds that create a cysteine-stabilized αβ motif [28]. In addition, these proteins lack a hydrophobic inner center, leading to a condensed knot-like structure. Some plant defensins have a fifth disulfide bond, which might be important for the stabilization of the protein core, and which allows variation in the surface loops [29]. Finally, the γ-core motif was proposed to be an essential region for the modes of action of the defensin MtDef4 of *Medicago truncatula* [30]. An insertion into target membranes has been proposed for some plant defensins, since high affinity binding sites exist [31], and since defensins can form weakly anion-selective channels [32].

Defensin genes of *Medicago truncatula* were found to be expressed in a variety of tissues and in response to different abiotic and biotic stimuli [33]. In the root nodule symbiosis, hundreds of root nodule-specific defensin-like proteins designated nodule cysteine-rich (NCR)-peptides act as effectors of bacteroid development [34, 35]. With respect to the AM symbiosis, Hanks *et al*. [33] were first to identify defensin genes induced in response to root colonization of *Medicago truncatula* by the AM-fungus *Glomus versiforme*, using a combination of transcript sequencing and real-time RT-PCR. Subsequently, several genome-wide transcriptomic studies confirmed that a family of defensin genes is activated during the AM symbiosis of *Medicago truncatula* [19, 36, 37; 38, 18] and for some, an arbuscule-correlated expression was demonstrated [37, 18].

In congruence with the significant impact of NCR-peptides on terminal bacteroid differentiation in legume root nodules [34, 35], defensins could have important functions for the morphology and life-cycle of arbuscules as well. It is thus the aim of this study to contribute to the functional characterization of mycorrhiza-dependent defensins of *M*. *truncatula*, based on a set of four AM-induced *MtDefMd* genes identified in expression profiling experiments [37, 38, 18]. Focusing on the two *MtDefMd* genes most strongly activated in a time-course of mycorrhization, we are able to show that their general and cell-specific expression is dependent on key genes of arbuscule formation *(MtRam1*) and function (*MtPt4*), respectively. Using translational fusions with subcellular fluorophore markers, we demonstrate that the *MtDefMd1* gene is not only specifically transcribed in arbuscule-containing cells, but that the encoded defensin is present during late restructuring processes of arbuscule-containing cells, providing novel insights into their transition into a post-symbiotic state.

## Materials and methods

### Bioinformatic analyses

Amino acid sequences of mature defensins were aligned using the following settings: Gap open cost: 10.00; gap extension cost: 5; Kyte-Doolittle window lenght 5; polarity logo. *RaptorX* [39] was used to build a three dimensional model of MtDefMds and AM-unrelated defensins. For this, the three-dimensional structures of HsAFP1 [40], RsAFP1 [41], major pollen allergen Art. v1 [42], and MtDef4 [30] were used as homology models. The coding sequences of *MtDefMd1* (Medtr.8g012805.1 in the *Medicago truncatula* genome [43, 44], Mtr.35854.1.S1_at in the *Medicago* Gene Expression Atlas (Benedito *et al*., 2008), *MtDefMd2* (Medtr.8g012835.1, Mtr.7210.1.S1_at), *MtDefMd3* (Medtr.8g012875, Mtr.3215.1.S1_at), and *MtDefMd4* (Medtr.8g012885, Mtr.31214.1.S1_at) were analyzed with *SignalP* [45], *Plant-mPloc* [46], and *SherLoc2* [47].

### Plant cultivars, fungal, and bacterial strains

*Medicago truncatula* Gaertn. Jemalong A17 (Thierry Huguet, INRA Toulouse, France) was used for all plant experiments. Sterile *Rhizophagus irregularis* DAOM197198 spores (PremierTech, Rivière-du-Loup, Canada) were applied as fungal inoculum. *Escherichia coli* DH5α mcr' [48] was used for cloning and the propagation of plasmids. Transgenic roots were induced by *Agrobacterium rhizogenes* Arqua1 [49].

### Cloning of promoter-*gusA*int fusions

Using genomic DNA of *M*. *truncatula* leaves, the promoters of *MtDefMd1 and MtDefMd2* (-1 to -1175 bp and -1 to -1457 bp relative to the translational start) were PCR-amplified using primers aaagaattcATTTGTCGTAAATAACCTTGC and aaaaagcttgCTTGCTTAATGTAAATGGAA (*MtDefMd1*) as well as aaacccgggCGCTTTTAGTTTTCGGTAGAT and aaacccgggGCTTGCTTAATGTAAATGGAA (*MtDefMd2*). Fragments were cleaved by *Sma*I and *Eco*RI/*Hin*dIII, respectively, and were cloned into pk18 [50]. The promoters were then subcloned into pGUS-INT [51], containing the *gusA*int reporter gene. Using *Spe*I, the transcriptional fusion was cleaved out, Klenow-blunted, and ligated into the *Sma*I-digested binary vector pRedRoot [52]. Promoter-*gusA*int fusions cloned in pRedRoot were transferred via electroporation into *A*. *rhizogenes* Arqua1.

### Cloning of knock-down and overexpression constructs

To obtain an RNAi construct targeting the highly similar *MtDefMd1* and *MtDefMd2* genes, the coding sequence of *MtDefMd1* was PCR-amplified from position +2. *attB* sites were added for cloning into the entry vector pDONR^TM^221 (Gateway®-System, Invitrogen, Karlsruhe, Germany), using the BP clonase reaction. The LR clonase reaction was used for cloning into the destination vector pK7GWIWG2(II)-*Q10*:DsRED [53].

To obtain an overexpression construct, the coding sequence of *MtDefMd1* (656 bp, containing introns) was PCR-amplified, cleaved with *Bam*HI and *Spe*I and ligated into vector *p9RFP* [54, 55] containing the *MtPt4* or *ubiquitin* promoters, respectively. *Spe*I and *Bam*HI recognition sites as well as 5 additional adenosin bases were added to the primers aaaaactagtATGGCTTCCTCTGCTCTTAAAT and aaaaggatccTTAGCAGTTGAAGTAACAGAAGCAAG.

### Cloning of constructs for subcellular localizations

A cassette of the native promoter and the coding sequence of *MtDefMd1* (-1 to -1175 bp; 656 bp coding region) was cloned into vector *p35S-OLI-mGFP6* [56]. This vector encodes an N-terminal GGRSPGGS oligopeptide (OLI) extension of mGFP6 that serves as a flexible linker to the protein of interest. The following cloning strategy were used: *Sph*I and *Kpn*I recognition sites were added to the primers for cloning the *MtDefMd1* promoter (aaagcatgcATTTGTCGTAAATAACCTTGCCT/ aaaggtaccGCTTGCTTAATGTAAATGGAAATG), and *Kpn*I and *Bgl*II were used for the coding sequence (aaaggtaccaaaaATGGCTTCCTCTGCTCTTA/aaaagatcttcctccGCAGTTGAAGTAACAGAAG), allowing the translation of an MtDefMd1-OLI-mGFP6 fusion protein under the control of the native *MtDefMd1* promoter instead of p35S. All primers were extended by 3 additional adenosin bases. The *MtDefMd1-OLI-mGFP6* fusion was released using *Eco*RI and *Hin*dIII. The fragment was blunted using Klenow polymerase and ligated into the blunted *Sal*I site of binary vector *pBIN*:*ER-ck* [57] expressing an *ER-CFP* marker. Finally, the fusion of the *MtBcp1* promoter and signal peptide region to the coding sequence of mCherry (*MtBcp1*_*SP*_*-mCherry*) was released from *pCMbB-TMEr* [58] using *Ecl*136II and *Sma*I. The insert was introduced into the *Sma*I site of the vector containing the *MtDefMd1-OLI-mGFP6* and *ER-CFP* fusions, resulting in a three-fluorophore vector designated *pBIN*:*ER-ck*:*MtDefMd1-OLI-mGFP6*:*MtBcp1*_*SP*_*-mCherry*. Vector pBIN:tp-gk [57] was used for tonoplast localization.

### Cultivation of *M*. *truncatula* plants

Plants were cultivated in a phytocabinet (Klimaschrank KPS 1700, Weisshaar, Bad Salzuflen, Germany) with 16 h/d light (Osram FLUORA L 18WI77 light tubes) at 22°C with a relative humidity of 60%. Plants were fertilized with ½ strength Hoagland's solution [59] containing 20 μM phosphate. The solution was prepared with deionised water; the pH was adjusted to 6.3 with H_2_SO_4_.

### Induction of transgenic *M*. *truncatula* roots and inoculation of *M*. *truncatula* with AM fungi

Transgenic *M*. *truncatula* roots were generated as described previously [60]. In short, *Agrobacterium rhizogenes* Arqua1 strains were grown for two days at 30°C on selective TY (0.5 g/l tryptone; 0.3 g/l yeast extract; 0.07 g/l CaCl_2_x2H_2_O) agar plates. Cells were resuspended in 4 ml PS buffer (40 mM Na_2_HPO_4_x2H_2_O, 85 mM NaCl, 17 mM KH_2_PO_4_; pH 7 adjusted with HCl). *M*. *truncatula* seedlings were moisturized and cleared of their testa. The bacterial solution was injected in the hypocotyl of the seedlings with a sterile syringe. Subsequently, seedlings were planted in sterile seramis® (Seramis GmbH, Mogendorf, Germany) and incubated over night at 18°C in the dark. Finally, they were transferred to the phytocabinet climate chamber. Transgenic hairy roots were screened and mycorrhized four to five weeks after germination. For most experiments, sterile *R*. *irregularis* spores were used for the inoculation of *M*. *truncatula* wild type and transgenic roots. 2000 spores were incubated with the shaded plant root for three hours in liquid. Subsequently, plants were potted in 9×9 cm pots with seramis (Seramis GmbH, Mogendorf, Germany) as a substrate. Remaining spores were pipetted on the roots. For RNAi experiments, *R*. *irregularis* inoculum from a leech preculture was used (Bettina Hause, IPB, Halle, Germany).

### Isolation of plant RNA from *M*. *truncatula* roots

RNA isolations were carried out using the RNeasy Plant Mini Kit (Qiagen, Hilden, Germany). ß-mercaptoethanol was added to the RLT buffer and 600 μl of this mix were added to each sample. Tissues were disrupted using the FastPrep®-24 (MP Biomedicals, Santa Ana, USA) for 5 times 30 s (6.5 m/s). The RNA concentrations were measured using a Nanodrop (Thermo Fisher Scientific, Langenselbold, Germany) and checked on agarose gels.

### Real-time RT-PCR

Real-time RT-PCR analyses were performed using the SensiFAST™ SYBR® No-ROX One-Step Kit (Qiagen, Hilden, Germany) with primers listed in the S1 Table. Primers were designed to match an annealing temperatures of 55°C and were tested for gene-specific amplifications. 5 ng of total RNA were used as a template in 20 μl for the mycorrhization time course, the *ram1-1* and the *MtDefMd1* overexpression experiment. Otherwise, 50 ng of total RNA were used. RT-PCR reactions followed a three-step cycling program: reverse transcription at 45°C for 10 min; polymerase activation at 95°C for 2 min; PCR amplification with 40 cycles at 95°C for 5 sec, 55°C for 10 sec, and 72°C for 8 sec.

For all genes, three technical replicates for each of 12 individual transgenic roots—if not stated differently—were measured. The housekeeping gene *MtTefα* was used for normalization. To relate gene expression to the degree of fungal colonization or the presence of active arbuscules, a ratio with *GiTubα* or *MtPt4* expression was calculated, respectively. The degree of fungal colonization was estimated by measuring the expression of the *GiTubα* gene encoding an alpha-tubulin [37], whereas the *MtPt4* transcript amount served as an estimate for the presence of active, phosphate-transporting arbuscules [61]. The mean and standard error of the mean of all biological replicates was calculated after normalization and was visualized, if not stated differently. Using MS Excel, two-tailed Student t-tests were performed to check differences in gene expression between mutant-, RNAi-, overexpression-, and wild type roots. To determine, if the expression of AM-related defensin and AM marker genes is congruent during the course of mycorrhization, Pearson correlation was calculated by using the MS Excel KORREL function for comparing the average gene expression levels at each time point.

### Histological studies

To study the activity of promoter-*gusA*int fusions, transgenic roots were incubated in GUS staining buffer described previously [51]. Cells with the strongest promoter activity were visible after 3 hours. After overnight staining, also cells with a weak promoter activity were stained. Finally, roots were rinsed with water.

To stain fungal structures for quantification of AM fungal matter via the gridline intersection method [62] and confocal microscopy, 1–2 cm root sections were incubated in 10% (w/v) KOH at 95°C for 7 minutes. Consecutively, the roots were washed three times with water and incubated in staining solution with 20 μg/ml Alexa-WGA Fluor 488 in 1x PBS (0.14 M NaCl, 2.7 mM KCl, 1 mM Na_2_HPO_4_x2H_2_O, 1.8 mM KH_2_PO_4_; pH 7.3) for 12–24 hours. Samples were protected from light. Finally, excessive dye was washed out with water. Mycorrhized root samples were randomly collected and pooled from each plant. Three pools of 6 root sections were photo-documented by confocal microscopy.

### Detection of reporter proteins via fluorescence microscopy

Transgenic *M*. *truncatula* roots were identified using a stereo microscope (Leica MZ10F, Sohns, Germany). 1–2 cm sections of mycorrhized roots were selected. For the localization of fluorescent reporter proteins, longitudinal root sections were cut by hand with a razor blade. These sections were transferred into a physiological buffer (39 mM Na_2_HPO_4_x2H_2_O, 86 mM NaCl, 22 mM KH_2_PO_4_; pH 7). GFP (500–520 nm), CFP (460–490 nm) and mCherry (599–621 nm) were detected in the inner root cortex using a hybrid detector and confocal microscopy (Leica TCS SP8 MP, Sohns, Germany). To evaluate, if the detected signals originate from the correct fluorophore, lambda-scans were performed in the range of 498–553 nm (mGFP6), 458–513 nm (CFP), and 575–635 nm (mCherry). Each lambda-scan contained eleven detection steps with 5 (mGFP6 and CFP), and 11 (mCherry) nm bandwidth, respectively. For promoter-GUS studies, an Eclipse TE2000-E inverse confocal laser scanning microscope (Nikon GmbH, Düsseldorf, Germany) and software EZ-C1 (Nikon GmbH, Düsseldorf, Germany) were used.

## Results

### Arbuscular mycorrhiza-related defensins differ from defence-related defensins

The four AM-related defensin genes *MtDefMd1*, *MtDefMd2*, *MtDefMd3*, and *MtDefMd4* (identifiers Mtr.35854.1.S1_at, Mtr.7210.1.S1_at, Mtr.3215.1.S1_at, and Mtr.31214.1.S1_at in the *Medicago* Gene Expression Atlas [63], respectively) were primarily identified based on their strong up-regulation in mycorrhizal roots [37].

**Fig 1:**
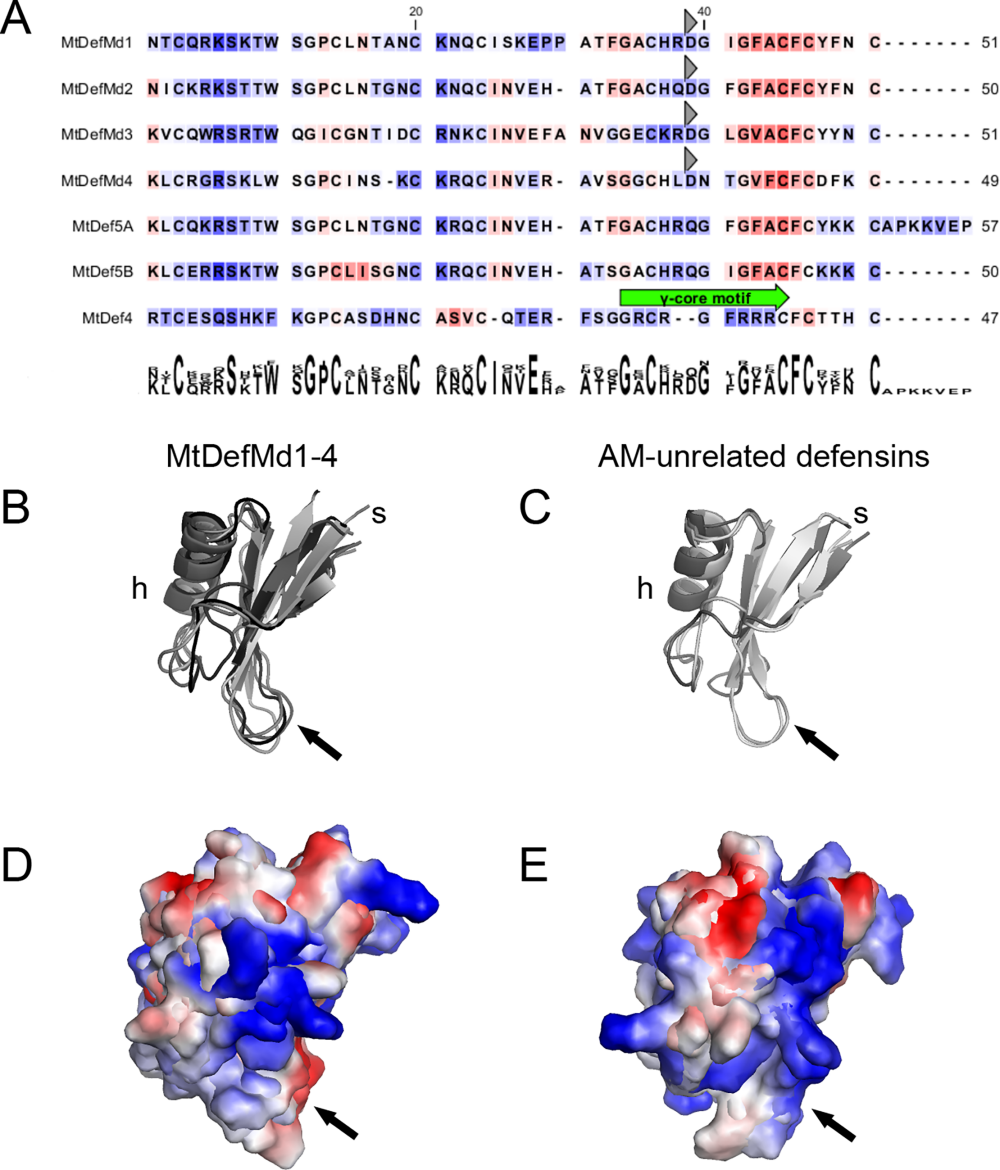
Sequence analyses of of AM-dependent defensins MtDefMd1-4 and AM-unrelated defensins. Secondary structures of MtDefMd1-4 and AM-unrelated defensin-like proteins of *M*. *truncatula* (A), a representation of their three-dimensional structures (B and C), as well as surface electrostatics (D and E) are shown. Predicted signal peptides were removed from the mature amino acid sequences. Consecutively, the defensins were aligned based on their secondary structures. Background colorisation of the amino acids (in A) indicate hydrophobicity in a scale from red to blue (red: high hydrophobicity). A conserved aspartic acid in the C-terminal region of MtDefMds is marked with a grey triangel. For the bi-domain defensin MtDef5, the domains MtDef5A (including a 7 amino acid linker towards the MtDef5B domain) and MtDef5B are shown. After modelling the three-dimensional structures of the MtDefMd1-4, MtDef4 and MtDef5A/B defensins, they were visualized (B and C) and their surface electrostatics were calculated (D and E). The region congruent to the γ-core motif is indicated with arrows. The following proteins were used for comparisons in addition to MtDefMd1-4: MtDef5 A [65], MtDef5 B [65]and MtDef4 [30].

In the deduced amino acid sequences of MtDefMd1-4, signal peptides were located up to the 29^th^ amino acid (Fig 1, S2 Table), suggesting that the defensins MtDefMd1-4 are secreted. Since it is known that the ER-Golgi network is redirected during the establishment of arbuscules [64], a targeting of MtDefMd1-4 to the periarbuscular space (PAS) in symbiotic cells is thus possible.

To investigate the secondary structures of the AM-dependent defensins MtDefMd1-4 in comparison to the AM-unrelated defensins MtDef4 [30] and MtDef5A/B [65], the amino acid sequences of the mature proteins were compared (Fig 1A). Subsequently, the three-dimensional structures of MtDefMd1-4 [based on HsAFP1, 40; RsAFP1, 41; HsAFP1, 40; and major pollen allergen Art. v1, 42], MtDef5A [based on HsAFP1, 40], MtDef5B [based on HsAFP1, 40], and MtDef4 [MtDef4, 30] were modeled and visualized (Fig 1B and 1C).

AM-dependent MtDefMds as well as AM-unrelated defensins are structurally conserved, being composed of an α-helix, three β-strands and the γ-core motif (Fig 1B and 1C). The four AM-related defensins are characterized by a hydrophobic C-terminus and an acidic amino acid (aspartic acid) at position 42 (Fig 1A). Although this discriminates them from the AM-unrelated defensin MtDef4 [30], both domains of the AM-unrelated defensin MtDef5A/B, which was shown to confer a broad antifungal activity [65], also display hydrophobic amino acids at the C-terminus (Fig 1A). Interestingly, some areas of the MtDefMd1-4 and AM-unrelated defensins exhibit a different distribution of cationic amino acids (Fig 1D and 1E) and the region corresponding to the γ-core motif, a variable loop essential for entry of MtDef4 in fungal cells [30], displays a differing pattern of surface electrostatics in AM-dependent and -unrelated defensins (Fig 1D and 1E).

In conclusion, MtDefMd proteins exhibit some differences to non-symbiotic defensins and are predicted to be secreted, suggesting a function at the interface between plants and AM fungi.

### *MtDefMd* transcription is upregulated in the course of mycorrhization

**Fig 2:**
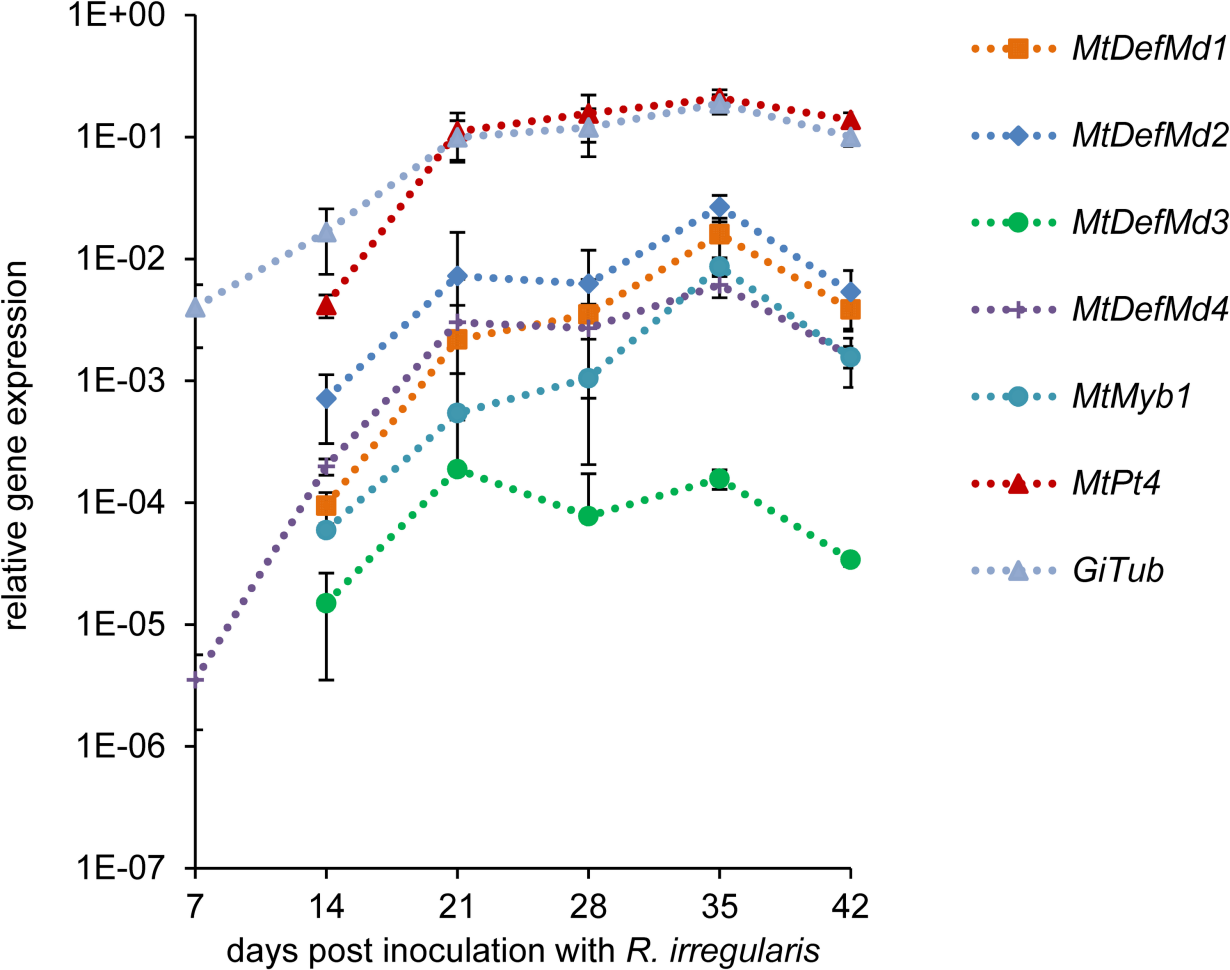
Relative expression of *MtDefMd1-4* and selected AM marker genes in *M*. *truncatula* roots in a time course of mycorrhization. Expression of *MtDefMd1-4*, the fungal *α*-tubulin gene *GiTubα* [36] as well as the *M*. *truncatula* AM marker genes *MtPt4* [66] and *MtMyb1* [17] is given in relation to the expression of *MtTefα*. Plant roots were harvested weekly from 7 to 42 days post inoculation with *R*. *irregularis*. Biological replicates were three pools of four plant roots per treatment. The standard deviation of the three biological replicates is given.

A mycorrhization time course was set to define the temporal regulation of *MtDefMd1-4* expression. *M*. *truncatula* plants were inoculated with *R*. *irregularis* spores using ½ strength Hoagland's solution containing 20 μM phosphate for fertilization, and the root system was harvested from 7 to 42 days post inoculation (dpi). The expression of *MtDefMd1-4*, a fungal *α*-tubulin (*GiTubα*) gene [36], and two *M*. *truncatula* AM marker genes (*MtPt4* [66], *MtMyb1* [17]) was measured via real-time RT-PCR (Fig 2), using the *MtTefα* gene encoding a translation elongation factor for normalization.

*MtDefMd1*, *MtDefMd2*, and *MtDefMd4* expression strongly correlated (r≥0.8) to that of all AM marker genes, while the correlation of *MtDefMd3* transcription was lower but still positive (r≥0.51, S3 Table). Transcription of the *MtDefMd* genes thus followed the colonization of plant roots with *R*. *irregularis*, estimated by the expression of the fungal and plant AM marker genes *GiTubα*, *MtPt4*, and *MtMyb1* (Fig 2). Whereas transcription of the fungal α-tubulin gene and *MtDefMd4* already rose from 7 dpi in a constant manner (Fig 2), expression of the other three *MtDefMd* genes as well as the AM markers genes *MtPt4* [66] and *MtMyb1* [17] strongly enhanced from 14 dpi on. It can be concluded that fungal mass enhanced from the start of inoculation, while arbuscules were effectively built from 14 dpi. Interestingly, at 42 dpi, the expression of all genes measured decreased, possibly due to a control of the degree of mycorrhization by the plant.

We conclude that on a whole-root level, the expression of the four AM-activated *MtDefMd* genes correlates with the expression of marker genes for root colonization and AM formation.

### *MtDefMd1* and *MtDefMd2* promoters are active in cells with fully developed arbuscules

**Fig 3:**
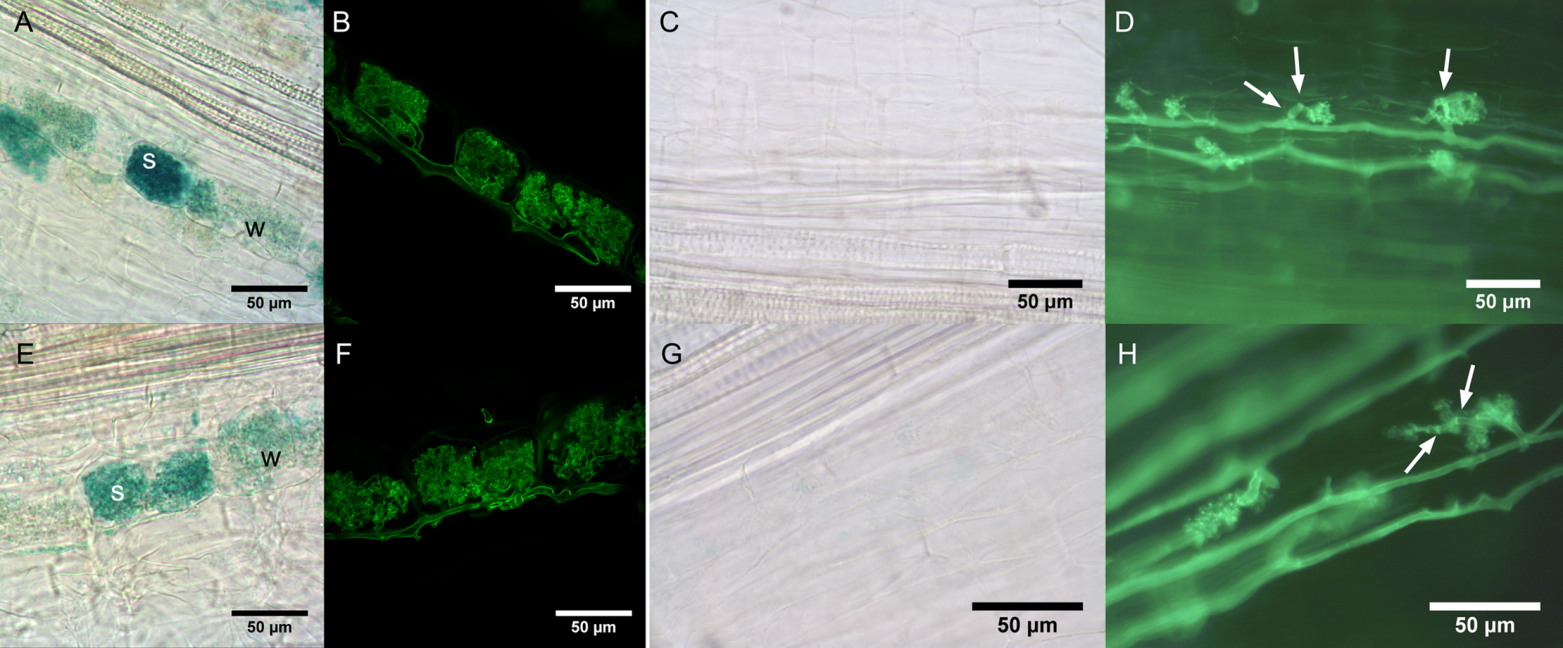
Histochemical localization of *MtDefMd1* and *MtDefMd2* promoter activities. Activities of *MtDefMd1* (A-D) and *MtDefMd2* (E-H) promoters were studied in transgenic, mycorrhized roots of *M*. *truncatula* A17 wild type (A, B, E, and F) and *pt4-2* roots (C, D, G, and H). Representative images of roots after 18 (A, B, E, and F) or 56 (C, D, G, and H) days post inoculation with *R*. *irregularis*. The GUS-stainings (A, C, E, and G) as well as the Alexa-WGA Fluor 488 stainings (B, D, F, and H) were performed over night. Septa are denoted by arrows. Abbreviations: w, cells with weak promoter activity; s, cells with strong promoter activity.

Since *MtDefMd1* and *MtDefMd2* are the defensin genes activated most strongly during the later colonization stages of *M*. *truncatula* roots with *R*. *irregularis* (Fig 2), we focused on these genes for further studies.Transcriptional fusions of the promoters of *MtDefMd1 and MtDefMd2* with the *gusA*int reporter gene were expressed in transgenic roots of *M*. *truncatula* A17 wild type to investigate their spacio-temporal expression during AM. Roots were mycorrhized for 18 dpi and analyzed via GUS and Alexa-WGA Fluor 488 stainings (Fig 3).

Activities of the *MtDefMd1* and *MtDefMd2* promoters were investigated from 7 to 56 dpi. In wild type roots, their activities varied from cell to cell, as shown for 18 dpi in (Fig 3A and 3E). While in pre-arbuscular stages and in cells with arbuscules appearing very young, no promoter activities were detected, GUS staining was present in cells with fully developed and apparently active arbuscules (Fig 3A and 3E). This indicates that the *MtDefMd1* and *MtDefMd2* promoters are activated in cells containing mature arbuscules.

To correlate *MtDefMd1* and *MtDefMd2* promoter activities with arbuscule development, the corresponding *gusA*int fusions were expressed in *pt4-2* mutants displaying premature arbuscule degeneration (PAD) [67]. Interestingly, there was no activity of the *MtDefMd1* and *MtDefMd2* promoters detectable even after 56 dpi (Fig 3C, 3D, 3G and 3H) and overnight staining. This observation corresponds to a downregulation of these genes in *pt4*-2 mutant in comparison to wild type roots [17].

We conclude that while *MtDefMd1* and *MtDefMd2* promoter activities are correlated with the presence of fully developed, active arbuscules, pre-mature arbuscule degradation does not involve transcription of these defensin genes. This suggests that MtDefMd defensins are not part of the PAD program activated in *pt4-2* mutants.

### *MtDefMd1 and MtDefMd2* expression is impaired in *ram1-1* mutants forming bird's-feet arbuscules

**Fig 4:**
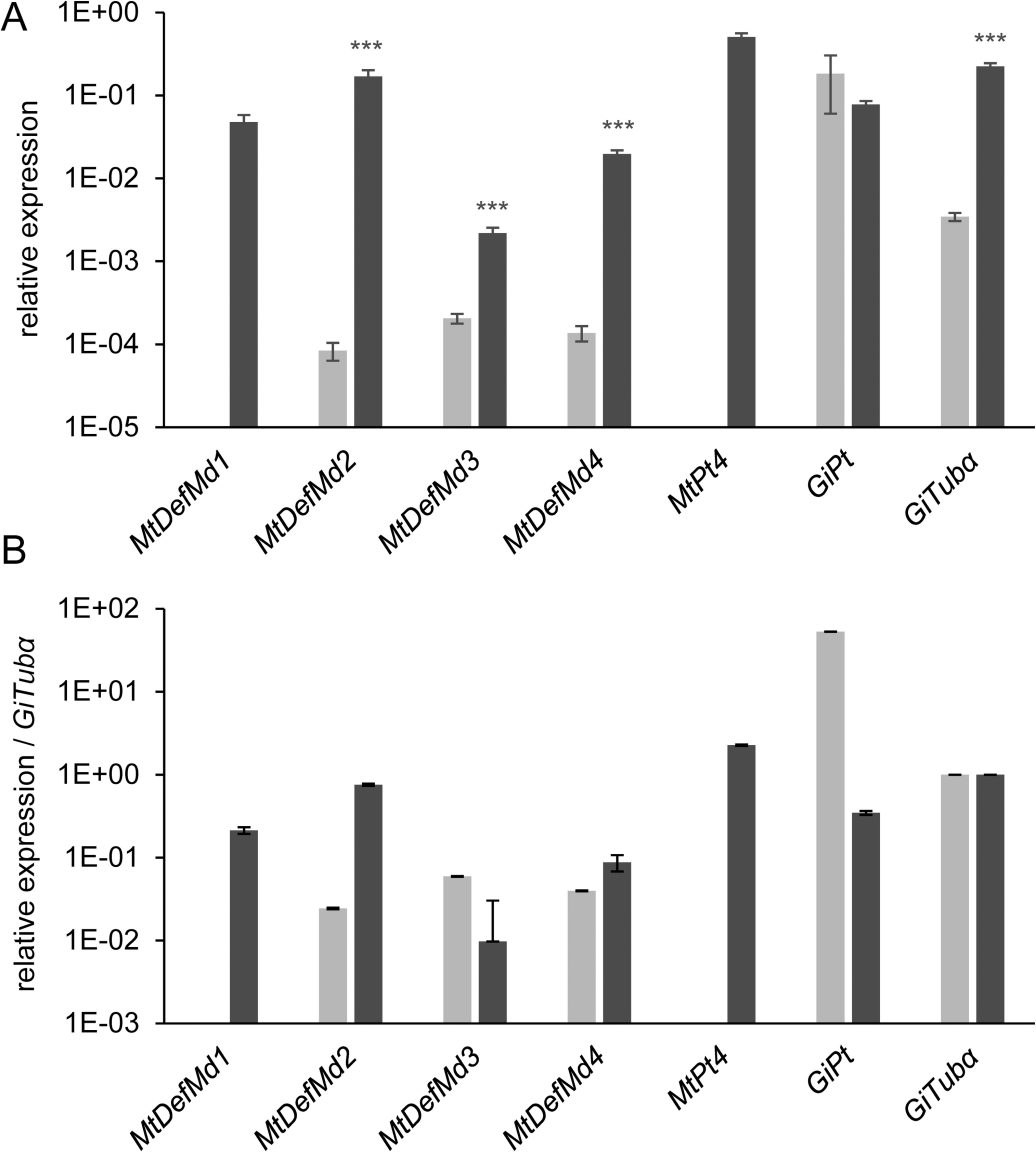
Relative expression of *MtDefMd* and selected AM-marker genes in mycorrhized *M*. *truncatula* A17 wild type and *ram1-1* roots. Transcript amounts are shown relative to *MtTefα* (A) and were additionally normalized by building a ratio with *GiTubα*-expression (B). *ram1-1* measurements are colored in light grey, the corresponding wild type measurements in dark grey. Roots were harvested at 36 days post inoculation with *R*. *irregularis*. n = 12 biological replicates, depicted is the standard error of the mean. Statistical significance: _***_ p≤0.0005.

To pinpoint the stage of arbuscule development, which coincides with *MtDefMd1* and *MtDefMd2* transcription, *ram1-1* mutants [68] were used. These mutants lack the trancription factor MtRam1, an important regulator that coordinates the further development of arbuscules post the bird's-feet stage by activating the transcription of a suite of AM-associated genes. To study *MtDefMd1* and *MtDefMd2* expression in relation to arbuscule maturation, mycorrhized *ram1-1* and wild type RNA samples were analyzed via real-time RT-PCR (Fig 4).

Whereas no *MtDefMd1* and *MtPt4* transcripts were detectable in mycorrhized *ram1-1* mutants, the expression of *MtDefMd2*, *MtDefMd3*, *MtDefMd4* and *GiTubα* appeared strongly reduced. Only a fungal phosphate transporter gene (*GiPt*) was not affected in expression. When normalizing gene expression to the level of root colonization by building a ratio with *GiTubα* expression, only the expression of *MtDefMd1*, *MtDefMd2*, and *MtPt4* appeared absent or strongly downregulated in *ram1-1* mutants (Fig 4B).

Based on these studies, the time point of MtDefMd1 and MtDefMd2 action can be placed in the arbuscule-containing cells, post the bird's-feet stage. This corresponds to our observation that the promoters of AM-related defensin genes are active in cells containing fully developed arbuscules.

### Knock-down of *MtDefMd1* and *MtDefMd2* does not affect expression of AM marker genes

Since *MtDefMd1* and *MtDefMd2* promoter activities were connected to fully developed arbuscules, we investigated, if their knock-down would affect arbuscule development. To reduce transcription of *MtDefMd1* and *MtDefMd2* simultaneously, an RNA interference (RNAi) construct targeting both genes was expressed in mycorrhized transgenic *M*. *truncatula* roots.

**Fig 5:**
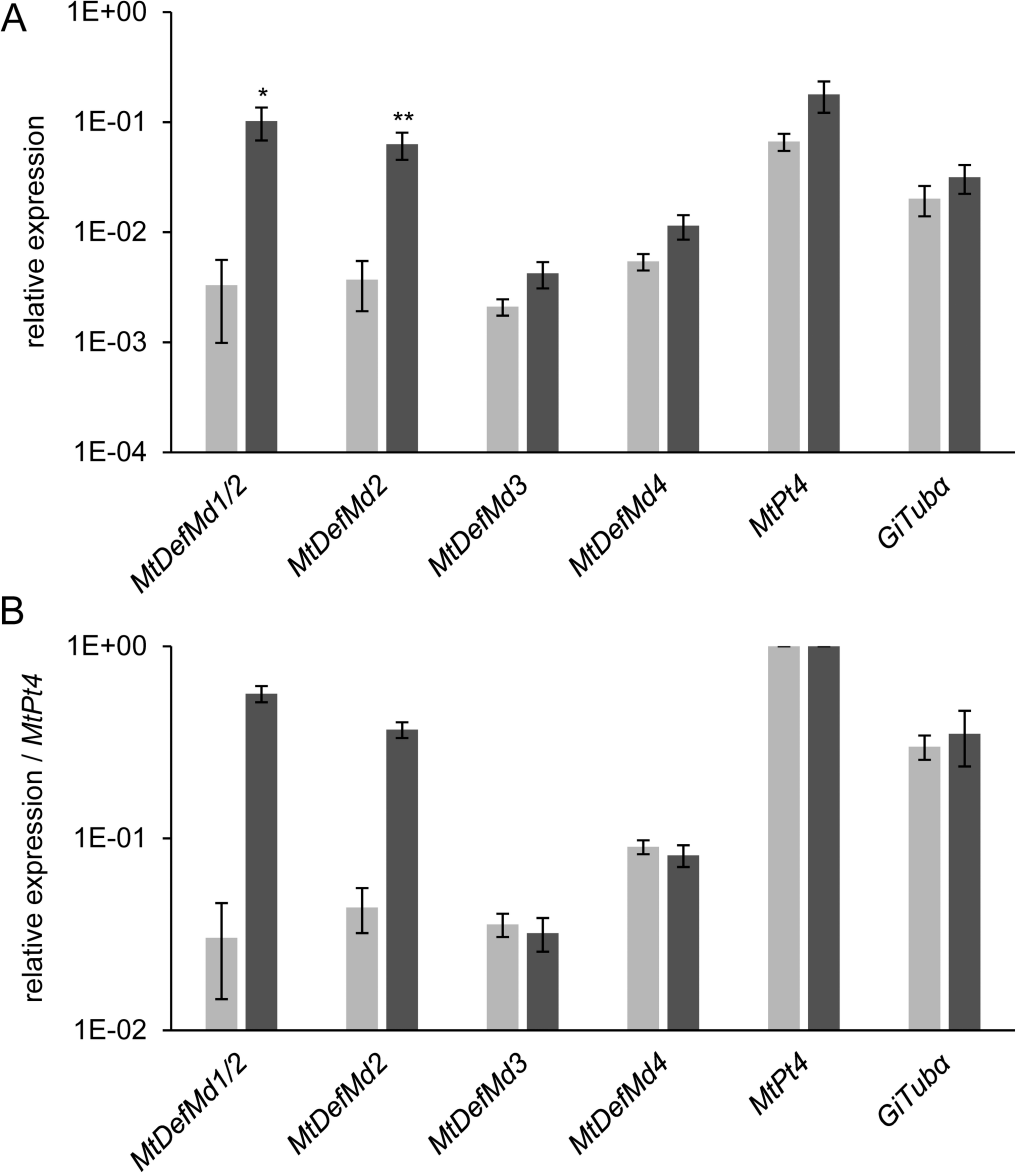
Relative expression of *MtDefMd* and selected AM marker genes in mycorrhized RNAi:MtDefMd1/2 and RNAi:*gusA*int transgenic control roots of *M*. *truncatula*. Transcript amounts are shown relative to *MtTefα* (A) and were additionally normalized by building a ratio to *MtPt4*-expression (B). Measurements from RNAi:MtDefMd1/2 roots are colored in light grey, corresponding RNAi:*gusA*int control measurements in dark grey. Roots were harvested at 28 days post inoculation with *R*. *irregularis*. n = 12 biological replicates, depicted is the standard error of the mean. Statistical significances: _*_ p≤0.05, _**_ p≤0.005.

Due to high similarities between the short *MtDefMd1* and *MtDefMd2* sequences, it was not possible to design gene-specific primers outside the region affected by the RNAi construct. Therefore, both genes were measured simultaneously in mycorrhized transgenic RNAi- and control roots (Fig 5). Since *MtDefMd2* expression alone was knocked down to 5.9%, the concomitant *MtDefMd1* and *MtDefMd2* reduction to appr. 3% indicated that similar to *MtDefMd2*, only residual amounts of *MtDefMd1* transcripts remained, confirming an efficient knock-down of both *MtDefMd1* and *MtDefMd2* expression (Fig 5A). In contrast, transcript amounts of *MtDefMd3*, *MtDefMd4*, *MtPt4*, and *GiTubα* did not differ significantly between RNAi- and control roots (Fig 5A and 5B). When normalized to the number of active arbuscules by building a ratio to *MtPt4*-expression (Fig 5B), the reduction of *MtDefMd1* and *MtDefMd2* transcript amounts remained very strong.

Since the severe knock-down of *MtDefMd1* and *MtDefMd2* did not lead to significant changes in *MtPt4* and *GiTubα* expression, we conclude that these *MtDefMd* genes are not essential predecessors for a successful fungal colonization and for the formation of active, phosphate-transporting arbuscules.

### Overexpression of *MtDefMd1* activates *MtDefMd3* and *MtRam1*

**Fig 6:**
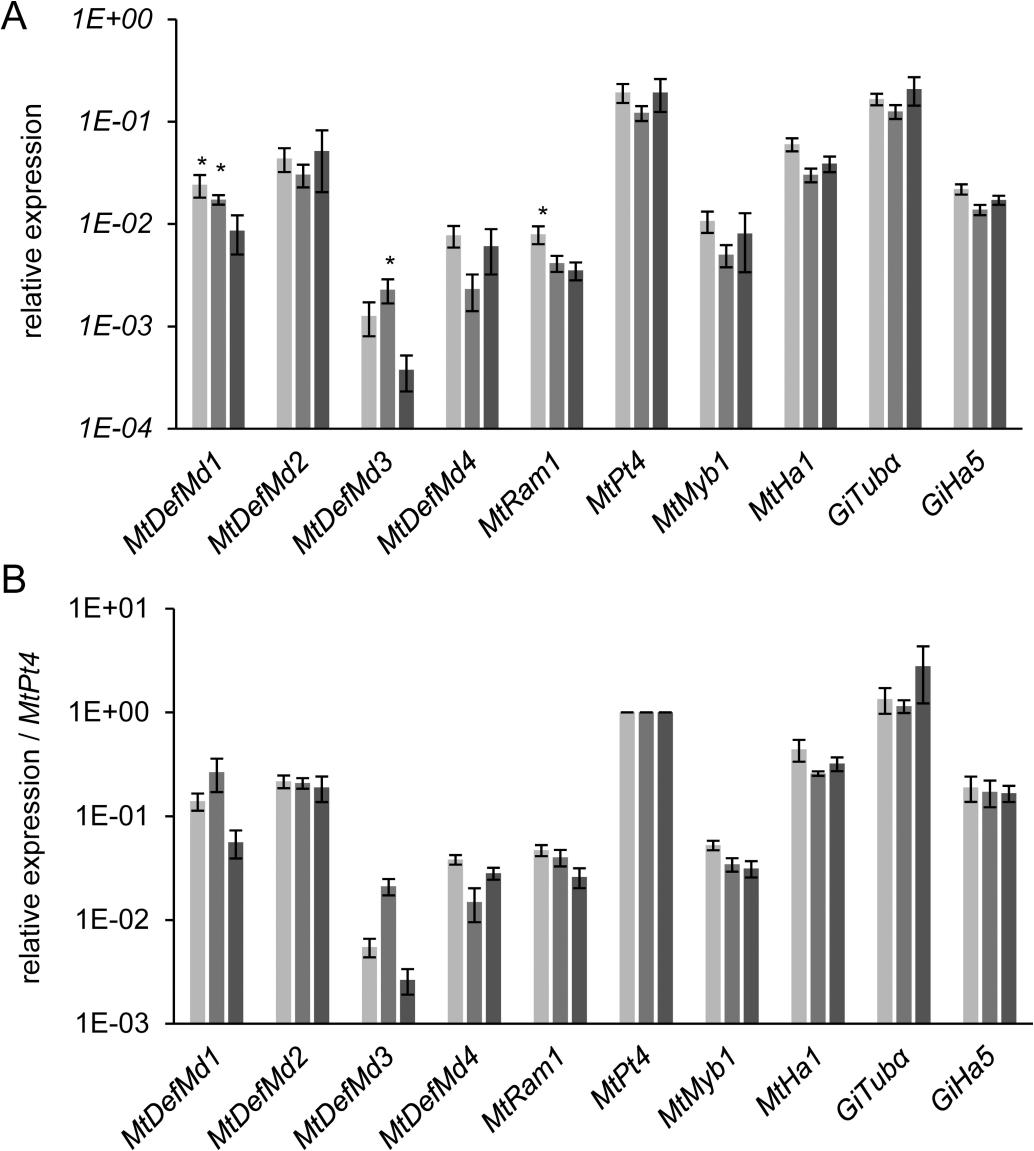
Relative expression of MtDefMd1 and selected AM marker genes in mycorrhized MtDefMd1-overexpression and pPT4:*gusA*int-expressing transgenic control roots of *M*. *truncatula*. Transcript amounts are shown relative to *MtTefα* (A) and were additionally normalized by building a ratio to *MtPt4*-expression (B). Measurements of pPT4:MtDefMd1 overexpression roots are coloured in light grey, measurements of pUbi:MtDefMd1 overexpression roots in medium grey, and measurements of pPT4:*gusA*int control roots in dark grey. Roots were harvested at 28 days post inoculation with *R*. *irregularis*. n = 12 biological replicates, depicted is the standard error of the mean. Statistical significance: * p≤0.05.

Due to the possibility that the four AM-related MtDefMds studied here and other yet undiscovered AM-related defensins act in parallel, it is difficult to elucidate their function by knocking down two of them. We thus decided to get insights into the role of defensins during AM by overexpressing the *MtDefMd1* gene in mycorrhized transgenic roots under the control of the *Ubiquitin*- and the *MtPt4*-promoter, mediating a general or a specific expression in arbuscule-containing cells, respectively (Fig 6).

Overexpression of *MtDefMd1* under control of the *MtPt4-* and the *Ubiquitin*-promoter led to a significant 2.8-fold and 2-fold overexpression of this defensin, respectively (Fig 6A). Whereas *MtDefMd3* was co-activated by *MtDefMd1* overexpression controlled by the *Ubiquitin*-promoter, expression of *MtDefMd2* and *MtDefMd4* was not affected (Fig 6A and 6B). Interestingly, transcription of *MtRam1* was 2.3-fold upregulated in case of an *MtDefMd1* overexpression driven by the *MtPt4* promoter. To further assess co-activation of *MtRam1* and *MtDefMd3*, transcript levels were normalized by building a ratio to *MtPt4* expression (Fig 6B), since the *MtDefMd1* and *MtDefMd2* promoters are specifically activated in the arbuscule-containing cells. Thereby, the overexpression of *MtDefMd1*, *MtRam1* and *MtDefMd3* was verified in relation to the formation of active arbuscules (Fig 6A and 6B).

### Knock-down of *MtDefMd1/2* transcription and overexpression of *MtDefMd1* has no significant impact on arbuscule size

**Fig 7:**
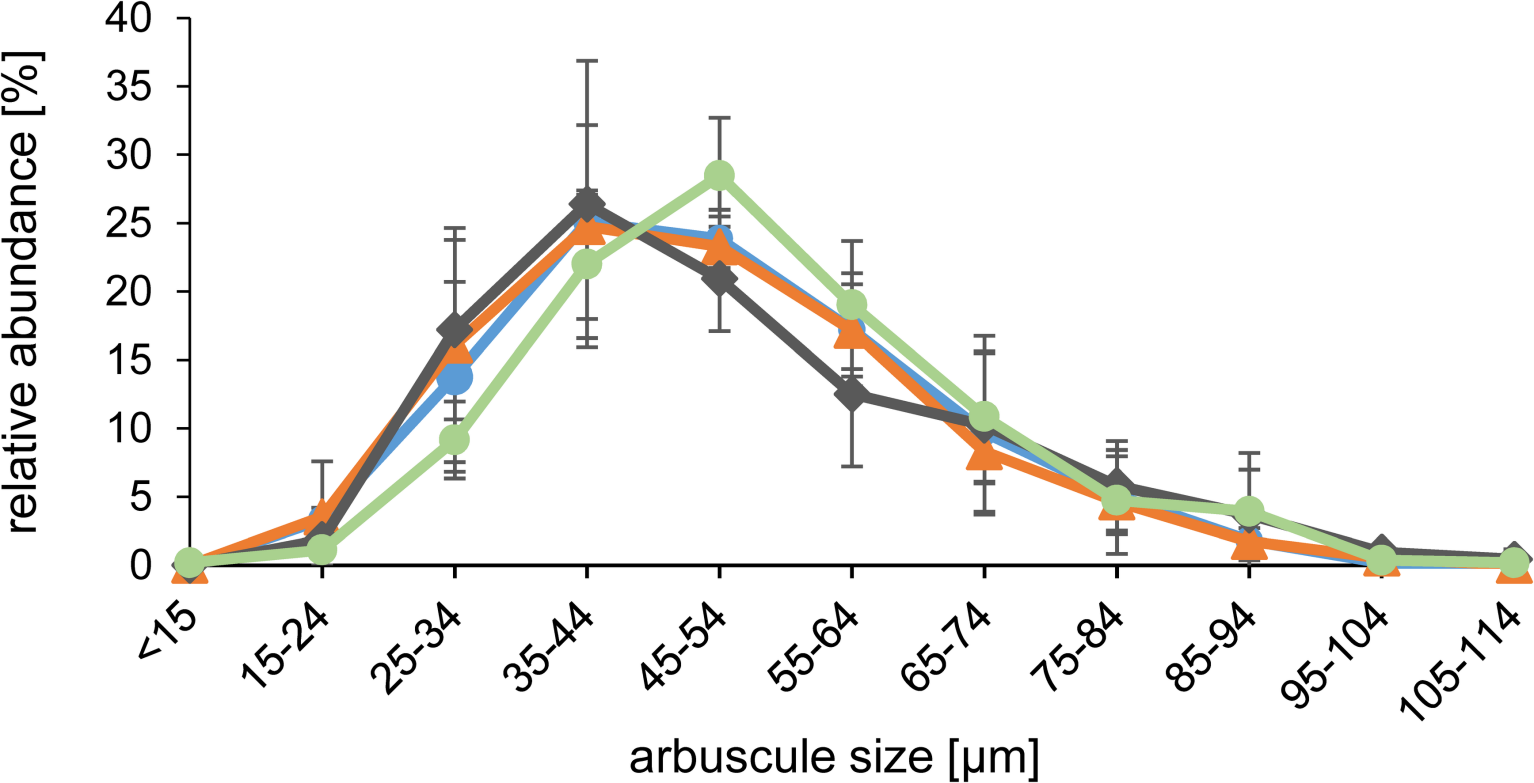
Size distribution of arbuscules in mycorrhized *M*. *truncatula MtDefMd1*-overexpression, *MtDefMd1/2*-knock-down, and pPT4:*gusA*int controls roots. Arbuscules were sorted into one of eleven size categories. In total, the size distribution of arbuscules in pUbi:MtDefMd1-overexpression (647 arbuscules), pPt4:MtDefMd1-overexpression (509 arbuscules), RNAi:MtDefMd1/2-knock-down (625 arbuscules), and pPt4:*gusA*int control roots (529 arbuscules) is depicted in orange, blue, green, and grey, respectively. Roots were harvested at 28 days post inoculation with *R*. *irregularis*. For each construct, three pools of root fragments, each pool being derived from four plants, were analysed. Depicted is the standard error of the mean.

To investigate, whether an *MtDefMd1/2* knock-down or the overexpression of *MtDefMd1* influences fungal colonization, phenotypical studies were performed in comparison to control roots. Due to the fact that on a whole-root level, no significant changes in the frequency of mycorrhization or the formation of arbuscles were observed (S4 Table, S5 Table), the distribution of arbuscule sizes was assessed via confocal microscopy of Alexa-WGA Fluor 488-stained root sections of mycorrhized RNAi:MtDefMd1/2 and *MtDefMd1*-overexpression in relation to pPt4:*gusA*int control roots (Fig 7).

Although the transcription factor gene *MtRam1* was upregulated by *MtDefMd1* overexpression (Fig 6), the sorting of arbuscules in different size categories indicated that similar to the whole-root level, there is no significant difference to the control roots (Fig 7). Thus, the global distribution of arbuscule sizes was congruent in *MtDefMd1* overexpression roots. Similarly, although a slight shift towards larger sizes was evident (Fig 7), no significant alteration of arbuscule sizes was recorded for the *RNAi*:*MtDefMd1/2* knock-down roots.

We conclude that neither an appr. 97% reduction of *MtDefMd1/2* transcription nor an up to 2.8-fold overexpression of *MtDefMd1* has a detectable effect on the steady-state distribution of arbuscule sizes.

### MtDefMd1 acts during late restructuring stages of arbuscule-containing cells

**Fig 8:**
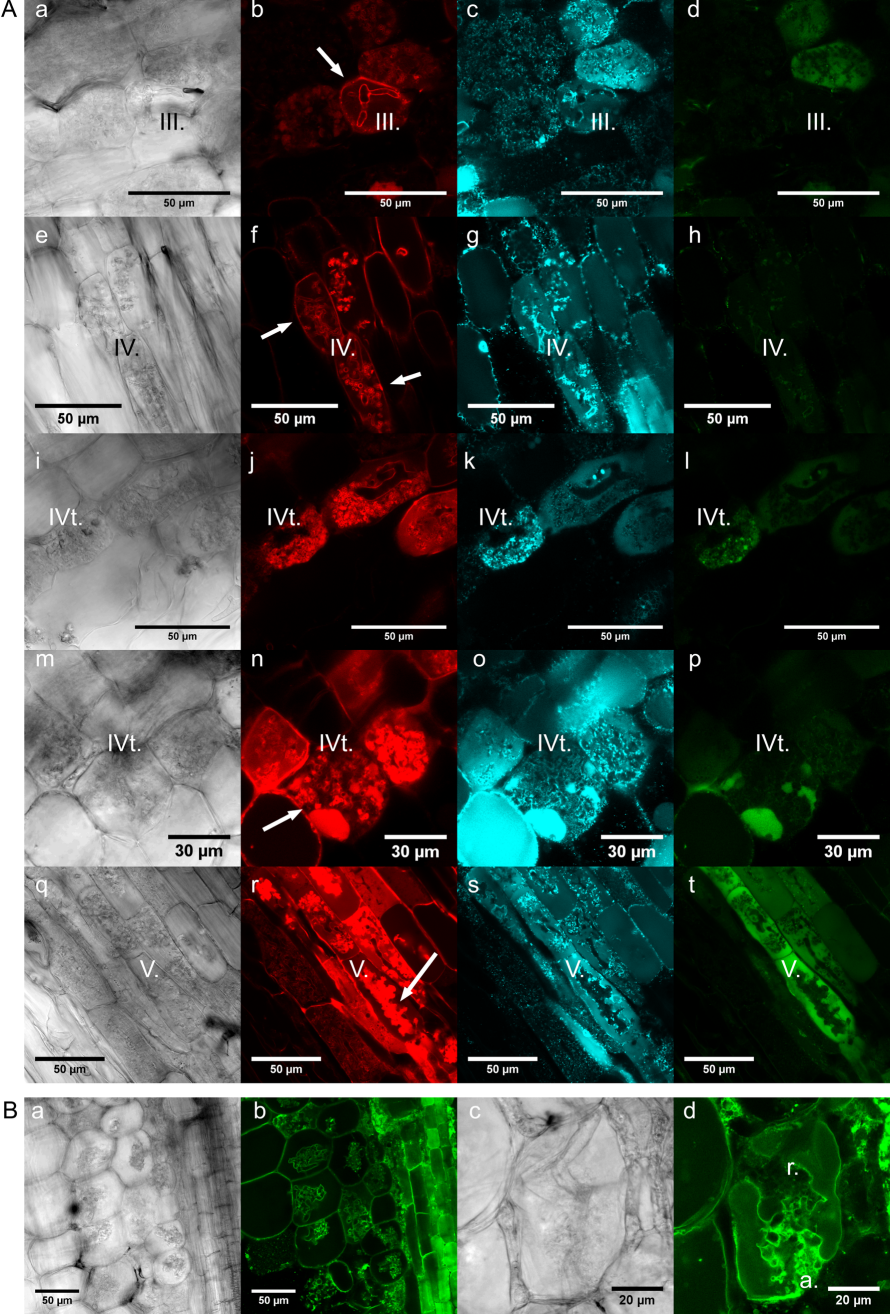
Localization of MtDefMd1-mGFP6 and additional fluorescence marker proteins in mycorrhized *M*. *truncatula* roots. Differential interference contrast micrographs of razor blade hand-cut sections of transgenic *M*. *truncatula* roots are shown (A; a, e, i, m, and q; B; a and c). Confocal microscopy was used to localize a fusion of the signal peptide of MtBcp1 with mCherry under the control of the *MtBcp1* promoter (A; b, f, j, n, and r), an ER-CFP fusion under the control of the 2x35S-promoter (A; c, g, k, o, and s), and an MtDefMd1-mGFP6 fusion under the control of the *MtDefMd1* promoter (A; d, h, i, p, and t). Additionally, a tonoplast membrane-directed GFP fusion under the control of a 2x35S-promoter was studied (B; b and d). Roots were mycorrhized with *R*. *irregularis* for six weeks. Arrows in the MtBcp1_SP_-mCherry micrographs indicate structures referred to in the text. Abbreviations: a., arbuscule branches; r., restructuring; III, bird's-feet arbuscule; IV, active arbuscule; IVt, fully developed arbuscule prior to collapsing; V, collapsed arbuscule.

From our promoter studies, we concluded that the two highly similar *MtDefMd1* and *MtDefMd2* genes are expressed in cells containing fully developed arbuscules. To monitor MtDefMd1 distribution and accumulation during the life-cycle of an arbuscule-containing cell, we designed a triple fluorophore reporter construct. First, we fused the MtDefMd1 coding region to mGFP6. In the resulting MtDefMd1-mGFP6 fusion, both regions are separated by a flexible linker. This modification enables free rotation between the two regions and was previously used to localize symbiosome-targeted proteins in the infected cells of root nodules [56]. To achieve a correctly regulated expression in the arbuscule-containing cells, the MtDefMd1-mGFP6 fusion was expressed under the control of the *MtDefMd1* promoter. To visualize protein secretion that is known to be transiently redirected towards the arbuscules [64], we introduced a constitutive endoplasmatic reticulum (ER) CFP-marker [57]. The signal peptide of MtBcp1 fused to mCherry [58] and expressed under the control of the *MtBcp1* [19] promoter, was used to define consecutive stages of the arbuscule life-cycle. MtBcp1 is present in the stem region of young and is more evenly distributed in the PAM of mature, fully developed arbuscules, thus allowing to define arbuscule stages [58]. All reporter fusions were concomitantly expressed in mycorrhized transgenic roots, and confocal microscopy was used to locate mCherry, CFP, and GFP fluorescence in the inner cortex (Fig 8).

In congruence to the MtBcp1-localisation reported by Ivanov and Harrison [58], the trunks of arbuscules in the bird's-feet stage (stage III.) were positive for MtBcp1_SP_-mCherry (Fig 8A, b). In addition, also the symplast membrane of cells in the symbiotic root tissues exhibited a certain level of mCherry fluorescence (Fig 8A, f). In mature, fully developed arbuscules, presumably active in metabolite exchange (stage IV.), MtBcp1_SP_-mCherry signals expanded to the whole PAM (Fig 8A, f and j).

Since the ER-marker was originally designed for the analysis of cell organelles in *Arabidopsis thaliana* [57], its functionality in *M*. *truncatula* had to be verified. It was expected that a spider web structure surrounding the nucleus, granular bodies at the edges of the cells as well as components of the cytoskeleton used to direct transport vesicles were stained. However, this was only the case for cells not containing arbuscules (Panel A in S1 Fig). In arbuscule-containing cells of the inner cortex, a close connection of ER-structures to the PAM (Fig 8A, c, g, k, o and s) and a CFP signal in the vacuole of GFP-positive cells (Fig 8A, o) was detected, indicating a massive reorganisation of vesicle traffic to or from the PAM.

MtDefMd1-GFP signals were only observed in a fraction of the arbuscule-containing cells (Fig 8A, I, p and t) and were not detected in cells prior to arbuscule branching (Fig 8A, d). In cases of low GFP signals, small dots and spheres in close proximity to the PAM of fully developed arbuscules were apparent (Fig 8A, l). These signals match the patterns of CFP-labeled ER-structures (Fig 8A, k, o and s) and while they are too large for being vesicles, they might be conglomerates of such. This points towards MtDefMd1 being part of the transportome redirected to the PAM. The MtDefMd1-mGFP6 signal increased strongly in cells with older, collapsing arbuscules (Fig 8A, p and t). These were characterized by structures appearing condensed and clumpy. In those cells, the mGFP6 signal gets very intensive and seems to be more and more present in the vacuole (Fig 8A, p and t).

To further investigate if the GFP pattern of intensively coloured cells matches restructuring processes of the vacuole during the AM symbiosis, transgenic roots expressing a tonoplast membrane GFP-marker [57], were mycorrhized and GFP-fluorescence was analyzed (Fig 8B, b and d). Whereas in non-mycorrhized roots, the space occupied by the cytoplasm, i.e. the volume close to the cell wall and cytoplasmic transvacuolar strains, are indirectly visualized (Panel B in S1 Fig), the space taken by the arbuscule is clearly defined by the surrounding GFP-labeled tonoplast (Fig 8B, b and d). The remaining space comprises the cytoplasm, the PAS and the symbiotic membranes from both organisms. Tonoplast membranes switch from a very close contact with arbuscule branches to a more loose one (Fig 8B, d, areas a and r). The morphology of cells exhibiting this phenomenon fits to those containing arbuscules in late stages of their life-cycle, characterized by a high level of MtDefMd1-mGFP6 (Fig 8A, p).

Our MtBcp1_SP_-mCherry, ER-CFP, and MtDefMd1-mGFP6 colocalization indicates that arbuscule structures undergo a transitional change between stage IV. (fully developed) and V. (degrading), which will be termed IVt. from now on. Once the distinct MtBcp1-mCherry signal in the arbuscule trunk (stage III.) extends towards the tips of the arbuscule branches (Fig 8A, j), the MtDefMd-mGFP6 signal starts to co-localize to these structures (Fig 8A, l; stage IV.t). Now, the distal tips of the arbuscule branches tend to get crescent-shaped or swollen, collapse (Fig 8A, j and n), and the space occupied by the arbuscule looks increasingly clumpy. Occasionally, the cell lumen contains non-fluorescent spheres of differing sizes, possibly representing partially degraded arbuscules (Panel A in S1 Fig). Strongly mGFP6-positive, most likely vacuolar compartments are increasingly created during stage IVt. and are abundant in stage V. (Fig 8A, p and t). The creation of fluorophore-positive vacuoles indicates a recycling of PAM-targeted vesicles and proteins, which were previously located in the perisymbiotic membrane. Since the increase of MtDefMd1-mGFP6 levels is strongest during this rather short and late stage of the arbuscule life-cycle (Fig 8A, l), it marks the beginning of the turnover from a symbiotic into a post-symbiotic cell.

## Discussion

AM-related defensins of the MtDefMd family can be distinguished from other antimicrobial peptides. First, the γ-core motif, which was vital for MtDef4 entry into *Fusarium graminearium* [30], is strikingly different in AM-related defensins. Second, a calculation of surface hydrophobicities identified a distinct pattern of hydrophobic amino acids and an aspartic acid residue close to the C-terminal region only for AM-related defensins, suggesting specific symbiotic targets for their function.

The shift of MtBcp1-mCherry from arbuscule trunks to the PAM allowed us not only to differentiate between pre-mature, fully developed, and collapsing arbuscules, but also to relate MtDefMd1-mGFP6 signals to these stages of arbuscule formation and degradation (Fig 8A, h to t). In contrast to MtBcp1-mCherry, MtDefMd1-mGFP6 signals were not detected prior to arbuscule branching (Fig 8A, d). Together with our observation that the strongest MtDefMd1-mGFP6 signals were found in cells with collapsing arbuscules and increasingly in vacuoles (Fig 8A, p and t), this indicates that MtDefMd1 functions during late stages of the arbuscule life-cycle, and here during the initiation of a regular turnover of symbiotic structures that involves massive restructuring of the PAM (Fig 8A, transition from h to l, l to p, and p to t). This is in line with our observation that the *MtDefMd1* and *MtDefMd2* promoters are activated in fully developed arbuscules and that the premature arbuscule degradation observed in *pt4-2* mutants does not involve *MtDefMd* genes.

Using *in planta* time-lapse imaging, it was shown that in contrast to their formation, arbuscule collapse is a rapid event [14]. Recent evidence indicates that the arbuscule degeneration program activated by MtMyb1 [17] plays an important role to initiate this step. In this light, it is conclusive that MtDefMd1 accumulation happens in a short period of time, defined by the IVt. stage (Fig 8A, i to p). The strong RNAi-mediated knock-down of *MtDefMd1* and *MtDefMd2* had no detectable impact on the function of arbuscules. Since the symbiotic nutrient exchange largely occurs before the onset of stage IVt, this could be expected. On the other hand, an *MtPt4*-promoter controlled overexpression of *MtDefMd1* led to higher *MtRam1* transcription, indicating that an enhanced level of *MtDefMd* transcripts can lead to differential expression of a key transcription factor that invokes a faster development of the arbuscule interface (MtRam1) [16].

Interestingly, nodule-specific defensins were found in *Alnus glutinosa* root nodules [69]. AG5, one of these, caused physiological changes of *Frankia* vesicles and increased their permeability [69], whereas four additional defensins displayed partly overlapping functions [70]. Apart from classical non-symbiotic and symbiotic defensins, more than 700 defensin-like peptides containing N-terminal signal peptides and conserved cysteine residues were identified in legume plants [26]. These nodule-specific cysteine rich (NCR) peptides either contain 4 or 6 cysteines, and some of them display remarkable similarities with AM-related defensins. NCR-peptides are targeted to symbiosomes via the secretory pathway [35, 71]. Inside the lumen of nitrogen-fixing bacteroids, multiple targets of NCR-peptides were observed, e.g. ribosomal proteins, cell cyle regulators, ATP synthases, nitrogenase, and components of the TCA cycle [72, 73]. In concert, the effects of hundreds of cell-specific NCR-peptides lead to terminal bacteroid differentiation in the indeterminate-type legume root nodules [74].

Vacuolar V-SNARE receptors are recruited in early steps of bacteroid senescence [75, 76]. Cargo vesicles originally addressed to the vacuole are now delivered to degrading symbiosome membranes, transforming the enclosed perisymbiotic space to an acidic lytic compartment [77, 78]. Fragmented vacuoles are adaptations of mature arbuscule-containing cells [79], so these processes are of interest for AM as well. In addition, it was reported that similar to the peribacteroid space, the periarbuscular space is acidic [80]. This environment might be decisive for a protonation of the aspartic acid residue in the characteristic C-terminal region of mycorrhiza-related defensins, and this might be important for their function.

The massive formation of fluorophore-positive vacuoles at a specific point of the arbuscule life-cycle (Fig 8A, p and t) probably reflects the recycling of PAM-derived fatty acids and proteins by the host. Presence of large amounts of MtDefMd1-mGFP6 was not only confined to a rather short, but also to a very late moment in the arbuscule life-cycle (stage IVt.; Fig 8l, p and t,), a time point matched by the accumulation of oil droplets [15]. Since AM fungi are oleogenic, the discovery that lipid dropleds coincide with collapsed arbuscular branches [15] opens new perspectives for MtDefMd function.

Studies on *MtRam1* and *MtRam2* mutants demonstrated that in addition to sugars, fatty acids from the host are supplied to intraradical fungal structures [81, 82], thus compensating the lack of fungal multidomain fatty acid synthases [15, 83]. This direct supply of monoacylglycerol is probably an important source for the synthesis of triaglycerol stored in lipid bodies of the intraradical mycelium [84, 15]. The transfer of fatty acids probably occurs during the active, nutrient-exchanging phase of the arbuscule. At a later time point, arbuscules are regularly degraded, and this process generates another source of lipids. Although it is currently unknown, what mechanisms for the attachment of defensins to fungal membranes are used [30], it is intriguing that the *Raphanus sativus* AFP2 and the *Dhahlia merckii* AMP1 defensins are known to bind sphingolipids [31, 85, 30]. Although MtDefMd defensins exhibit some differences to non-symbiotic defensins, they are likely to have direct antifungal activity like other cationic, AM-unrelated defensins. In particular, AM-dependent defensins including their γ-core motifs share significant homologies with the recently published bi-domain MtDef5A/B, which exhibits potent antifungal activity *in vitro* [65]. Interestingly, MtDef5A/B has been shown to bind several phospholipids *in vitro*, including phosphatidylinositol monophosphates (PIP) and PIP2 [65]. It is thus likely that MtDefMd1 and MtDefMd2 also bind to phospholipids and this binding might be important for their biological function during later stage of the AM symbiosis. Taking the peak of MtDefMd1-mGFP6 synthesis in a short and late period of the arbuscule life-cycle into consideration, it is tempting to hypothesize that MtDefMd defensins bind specific lipid compounds during the massive digestion of degrading arbuscules, containing a significant amount of membrane lipids. During this process, these lipids might act as carriers for an endocytic incorporation of MtDefMd proteins, thereby limiting excessive fungal access to host lipids due to a potential toxicity of the defensins at high concentrations. This toxicity can e.g. be mediated via Ca^2+^-influx, K^+^-efflux, and a loss of membrane potential [86, 87, 88]. Since phytopathogenic fungi are supplied with host fatty acids as well, and reduced fatty acid synthesis impairs infections [82], mechanisms hindering the loss of plant fatty acids are an important mode of control.

The quick and strong accumulation of MtDefMd1 marks the initiation of a turnover in arbuscule-containing cells, ultimately leading to the post-symbiotic formation of cortical cells ready to be re-colonized. This transition requires not only transcriptional changes and cellular restructuring, but also antimicrobial treatments. Specifically, fungal structures collapse during the arbuscule degeneration initiated by MtMyb1 [17], while the now abandoned symbiotic market place is enriched with proteins and lipids of symbiotic membranes [14]. It might thus be beneficial for the plant to recycle such molecules by redirecting them to the vacuole that grows due to the fusion of initially fragmented areas. Absence of *MtDefMd1* and *MtDefMd2* transcription in *pt4-2* and *ram1-1* mutants has thus to be expected, since in defect or prematurely degraded arbuscules, no expanded symbiotic interface exists, where lipids need to be reconquered.

Due to the multiplity of their modes of action, functional assessments of defensin-like proteins are challenging. We here show that *MtDefMd1*, a member of the newly described *MtDefMd* family of mycorrhiza-activated defensins, is not only specifically transcribed during AM interactions, but is present during the restructuring processes of arbuscule-containing cells. Its presence at this defined, late stage of the arbuscule life-cycle might be a hallmark of the regular transition from symbiotic to post-symbiotic cells. Since the timing of defensin presence described here coincides with the reported accumulation of AM-fungal lipids [15], an association of AM-related defensins with these molecules is a starting point for furthers studies of MtDefMd function in the AM symbiosis.

## Supporting information

S1 Fig Localization of MtDefMd1-mGFP6 in mycorrhized *M*. *truncatula* roots expressing a multi-fluorophore reporter construct and a tonoplast marker. Confocal micrographs of razor blade hand-cuttings of transgenic *M*. *truncatula* roots. The roots express an MtDefMd1-mGFP6 fusion under the control of the native promoter (A; d and g), an ER-CFP fusion under the control of the 2x35S-promoter (A; c and f), and a fusion of the signal peptide of MtBcp1 with mCherry under the control of the native promoter (A; b). Additionally, a tonoplast membrane directed GFP fusion under the control of a 2x35S-promoter (B, b) is shown. Differential interference contrast (DIC) micrographs are shown for each root section (A; a and e; B, a). Roots were mycorrhized *with R*. *irregularis* for six weeks.
(TIF)

S1 Table Primers used in real-time RT-PCR experiments. (DOCX)

S2 Table Properties and cleavage sites of the MtDefMd signal peptides predicted by SignalP. (DOCX)

S3 Table Correlation of *MtDefMd* and AM marker gene expression in the course of mycorrhization. (DOCX)

S4 Table Percentage of colonized and arbuscule-containing areas in mycorrhized *Medicago truncatula MtDefMd1*-overexpression (pPt4:MtDefMd1, pUbi:MtDefMd1) and pPt4:*gusA*int controls roots. (DOCX)

S5 Table Percentage of colonized and arbuscule-containing areas in mycorrhized *Medicago truncatula* MtDefMd1/2-knock-down (RNAi:MtDefMd1/2) and RNAi:*gusA*int control roots. (DOCX)

S6 Table Underlying data points for the size distribution of arbuscules in mycorrhized *Medicago truncatula MtDefMd1*-overexpression (pPt4:MtDefMd1, pUbi:MtDefMd1), *MtDefMd1/2*-knock-down (RNAi:MtDefMd1/2), and pPT4:*gusA*int controls roots. (DOCX)
