# Supplementary figures and images for: The mycorrhiza-dependent defensin MtDefMd1 of Medicago truncatula acts during the late restructuring stages of arbuscule-containing cells
Source: PLoS One. 2018 Jan 25;13(1):e0191841. doi: 10.1371/journal.pone.0191841 (PMC5784984; doi:10.1371/journal.pone.0191841)

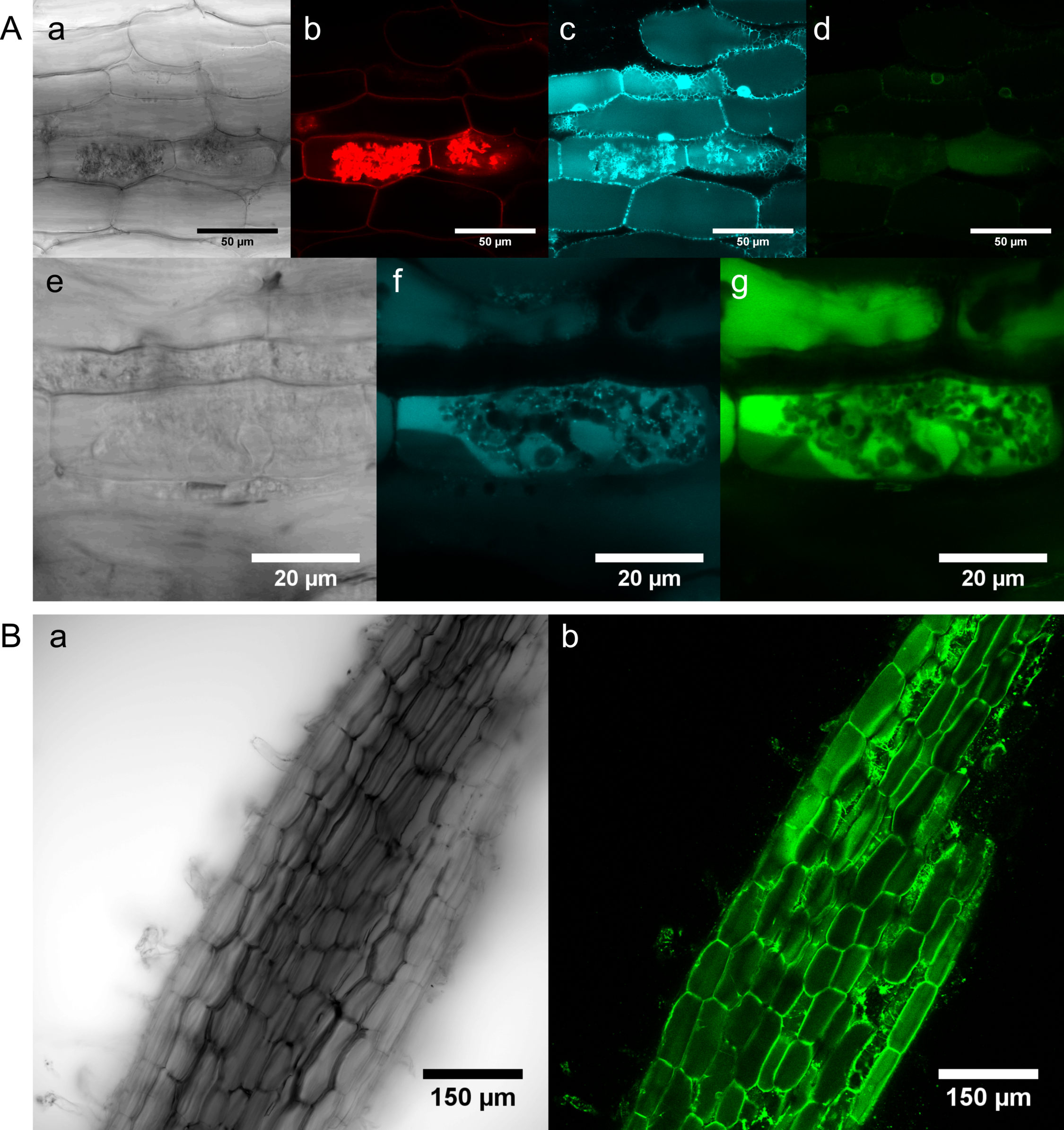

Supplement: S1 Fig — Confocal micrographs of razor blade hand-cuttings of transgenic M. truncatula roots. The roots express an MtDefMd1-mGFP6 fusion under the control of the native promoter (A; d and g), an ER-CFP fusion under the control of the 2x35S-promoter (A; c and f), and a fusion of the signal peptide of MtBcp1 with mCherry under the control of the native promoter (A; b). Additionally, a tonoplast membrane directed GFP fusion under the control of a 2x35S-promoter (B, b) is shown. Differential interference contrast (DIC) micrographs are shown for each root section (A; a and e; B, a). Roots were mycorrhized with R. irregularis for six weeks. (TIF) [file pone.0191841.s001.tif]
