# Supplementary material for: The mycorrhiza-dependent defensin MtDefMd1 of Medicago truncatula acts during the late restructuring stages of arbuscule-containing cells
Source: PLoS One. 2018 Jan 25;13(1):e0191841. doi: 10.1371/journal.pone.0191841 (PMC5784984; doi:10.1371/journal.pone.0191841)
Supplement: S1 Table — (DOCX) [file pone.0191841.s002.docx]

**S1 Table. Primers used in real-time RT-PCR experiments.**

| **Gene** | **Identifier*** | **Primers** |
| --- | --- | --- |
| *MtDefMd1* | Medtr8g012805.1 | gcttcctctgctcttaaata/tgttgccggtggttccttac |
| *MtDefMd2* | Medtr8g012835.1 | aaaggaaaagcacaacatgg/cagaagcaagcaaatccaaa |
| *MtDefMd1/2* | Medtr8g012805.1/Medtr8g012835.1 | gggacattaagcaagcatg/tcacattgcttgagcagaaa |
| *MtDefMd3* | Medtr8g012875.1 | tgctcgtaaattcctcacaa/cttcgccattgacaaacttt |
| *MtDefMd4* | Medtr8g012885.1 | ttgtagagggagaagcaaac/ccagtgttatcaaggtgaca |
| *MtTefα* | Medtr6g021805.1 | aagctaggaggtattgacaag/actgtgcagtagtacttggtg |
| *MtPt4* | Medtr1g028600.1 | tcgcgcgccatgtttgttgt/gcgaagaagaatgttagccc |
| *MtMyb1* | Medtr7g068600.1 | TACTGCCAAATTTCTGTTCTA/GGATTGTGTTTTAAAGGATTC |
| *MtRam1* | Medtr7g027190.1 | CATTACTACTCCGCAATTTTC/CAACAAACAACCTTTATCCTC |
| *MtHa1* | Medtr8g006790.1 | aggttctaacatctattggga/gacaacttatgtgaaccaatg |
| *GiHa5* | GQ205019.1 | aaattacggtttcttctgcac/tggaacatcttccttcatttca |
| *GiPt* | AY037894.1 | aacacgatgtcaacaaagcaac/aagaccgattccataaaaagca |
| *GiTubα* | GW088233.1 | tgtccaaccggttttaaagt/aaagcacgtttggcgtacat |

* From the *Medicago truncatula* genome 4.0 or GenBank accesssion numbers, respectively.
