# Supplementary material for: The mycorrhiza-dependent defensin MtDefMd1 of Medicago truncatula acts during the late restructuring stages of arbuscule-containing cells
Source: PLoS One. 2018 Jan 25;13(1):e0191841. doi: 10.1371/journal.pone.0191841 (PMC5784984; doi:10.1371/journal.pone.0191841)
Supplement: S2 Table — (DOCX) [file pone.0191841.s003.docx]

**S2 Table. Properties and cleavage sites of the MtDefMd signal peptides predicted by SignalP.**

| **Defensin** | **Cleavage site*** | **D** | **D-cutoff** | **Transmembrane region** |
| --- | --- | --- | --- | --- |
| MtDefMd1 | VQG-NT | 0.853 | 0.450 | No |
| MtDefMd2 | VQG-NI | 0.892 | 0.450 | No |
| MtDefMd3 | VEA-KV | 0.877 | 0.450 | No |
| MtDefMd4 | VQA-KL | 0.919 | 0.450 | No |

* Cleavage is between positions 29 and 30 in each case.
