# Supplementary material for: The mycorrhiza-dependent defensin MtDefMd1 of Medicago truncatula acts during the late restructuring stages of arbuscule-containing cells
Source: PLoS One. 2018 Jan 25;13(1):e0191841. doi: 10.1371/journal.pone.0191841 (PMC5784984; doi:10.1371/journal.pone.0191841)
Supplement: S3 Table — (DOCX) [file pone.0191841.s004.docx]

**S3 Table. Correlation of *MtDefMd* and AM marker gene expression in the course of mycorrhization.**

|  | *MtDefMd1* | *MtDefMd2* | *MtDefMd3* | *MtDefMd4* |
| --- | --- | --- | --- | --- |
| *MtDefMd1* | 1.00 | 0.99 | 0.57 | 0.92 |
| *MtDefMd2* | 0.99 | 1.00 | 0.68 | 0.96 |
| *MtDefMd3* | 0.57 | 0.68 | 1.00 | 0.82 |
| *MtDefMd4* | 0.92 | 0.96 | 0.82 | 1.00 |
| *MtMyb1* | 0.99 | 0.98 | 0.51 | 0.88 |
| *MtPt4* | 0.80 | 0.81 | 0.67 | 0.89 |
| *GiTubα* | 0.87 | 0.89 | 0.74 | 0.95 |
