# Supplementary material for: The mycorrhiza-dependent defensin MtDefMd1 of Medicago truncatula acts during the late restructuring stages of arbuscule-containing cells
Source: PLoS One. 2018 Jan 25;13(1):e0191841. doi: 10.1371/journal.pone.0191841 (PMC5784984; doi:10.1371/journal.pone.0191841)
Supplement: S4 Table — (DOCX) [file pone.0191841.s005.docx]

**S4 Table. Percentage of colonized and arbuscule-containing areas in mycorrhized *Medicago truncatula* *MtDefMd1*-overexpression (pPt4:MtDefMd1, pUbi:MtDefMd1) and pPt4:*gusA*int controls roots.**

| Construct | Plants [n] | Grid line intersections | Intersections with fungal structures [%] | Intersections with arbuscules [%] |
| --- | --- | --- | --- | --- |
| pPt4:MtDefMd1 | 6 | 300 | 60* | 46* |
| pUbi:MtDefMd1 | 12 | 300 | 71* | 59* |
| pPt4:*gusA*int | 9 | 300 | 64 | 54 |

* No significant difference to pPt4:*gusA*int control roots
