# Supplementary material for: The mycorrhiza-dependent defensin MtDefMd1 of Medicago truncatula acts during the late restructuring stages of arbuscule-containing cells
Source: PLoS One. 2018 Jan 25;13(1):e0191841. doi: 10.1371/journal.pone.0191841 (PMC5784984; doi:10.1371/journal.pone.0191841)
Supplement: S5 Table — (DOCX) [file pone.0191841.s006.docx]

**S5 Table. Percentage of colonized and arbuscule-containing areas in mycorrhized *Medicago truncatula* MtDefMd1/2-knock-down (RNAi:MtDefMd1/2) and RNAi:*gusA*int control roots.**

| Construct | Plants [n] | Grid line intersections | Intersections with fungal structures [%] | Intersections with arbuscules [%] |
| --- | --- | --- | --- | --- |
| RNAi:MtDefMd1/2 | 5 | 301 | 52* | 41* |
| RNAi:*gusA*int | 8 | 308 | 59 | 57 |

* No significant difference to RNAi:*gusA*int control roots
