# Supplementary material for: The mycorrhiza-dependent defensin MtDefMd1 of Medicago truncatula acts during the late restructuring stages of arbuscule-containing cells
Source: PLoS One. 2018 Jan 25;13(1):e0191841. doi: 10.1371/journal.pone.0191841 (PMC5784984; doi:10.1371/journal.pone.0191841)
Supplement: S6 Table — (DOCX) [file pone.0191841.s007.docx]

**S6 Table. Underlying data points for the size distribution of arbuscules in mycorrhized *Medicago truncatula* *MtDefMd1*-overexpression (pPt4:MtDefMd1, pUbi:MtDefMd1), *MtDefMd1/2*-knock-down (RNAi:MtDefMd1/2), and pPt4:*gusA*int controls roots.**

| **Construct expressed in transgenic roots** | **Arbuscule size category** | **Arbuscules per category** | | | **Relative amount of arbuscules per category** | | | **Mean of relative amounts** | **Standard deviation** | **t-test* vs pPt4:*gusA*int** |
| --- | --- | --- | --- | --- | --- | --- | --- | --- | --- | --- |
|  |  | **I** | **II** | **III** | **I** | **II** | **III** |  |  |  |
| pPt4:MtDefMd1 | <15 | 0 | 0 | 0 | 0 | 0 | 0 | 0.00 | 0.00 | - |
| pPt4:MtDefMd1 | 15-24 | 5 | 6 | 11 | 2.49 | 3.21 | 4.25 | 3.31 | 0.88 | 0.070 |
| pPt4:MtDefMd1 | 25-34 | 17 | 21 | 56 | 8.46 | 11.23 | 21.62 | 13.77 | 6.94 | 0.566 |
| pPt4:MtDefMd1 | 35-44 | 38 | 44 | 85 | 18.91 | 23.53 | 32.82 | 25.08 | 7.09 | 0.866 |
| pPt4:MtDefMd1 | 45-54 | 52 | 45 | 56 | 25.87 | 24.06 | 21.62 | 23.85 | 2.13 | 0.311 |
| pPt4:MtDefMd1 | 55-64 | 43 | 32 | 34 | 21.39 | 17.11 | 13.13 | 17.21 | 4.13 | 0.292 |
| pPt4:MtDefMd1 | 65-74 | 30 | 20 | 9 | 14.93 | 10.70 | 3.47 | 9.70 | 5.79 | 0.921 |
| pPt4:MtDefMd1 | 75-84 | 10 | 15 | 6 | 4.98 | 8.02 | 2.32 | 5.10 | 2.85 | 0.796 |
| pPt4:MtDefMd1 | 85-94 | 5 | 4 | 2 | 2.49 | 2.14 | 0.77 | 1.80 | 0.91 | 0.397 |
| pPt4:MtDefMd1 | 95-104 | 1 | 0 | 0 | 0.50 | 0 | 0 | 0.17 | 0.29 | 0.042 |
| pPt4:MtDefMd1 | 105-114 | 0 | 0 | 0 | 0 | 0 | 0 | 0.00 | 0.00 | 0.374 |
| pUbi:MtDefMd1 | <15 | 0 | 0 | 0 | 0 | 0 | 0 | 0.00 | 0.00 | - |
| pUbi:MtDefMd1 | 15-24 | 15 | 5 | 0 | 7.98 | 2.59 | 0 | 3.52 | 4.07 | 0.522 |
| pUbi:MtDefMd1 | 25-34 | 41 | 39 | 8 | 21.81 | 20.21 | 6.25 | 16.09 | 8.56 | 0.865 |
| pUbi:MtDefMd1 | 35-44 | 42 | 52 | 32 | 22.34 | 26.94 | 25.00 | 24.76 | 2.31 | 0.805 |
| pUbi:MtDefMd1 | 45-54 | 39 | 48 | 31 | 20.74 | 24.87 | 24.22 | 23.28 | 2.22 | 0.408 |
| pUbi:MtDefMd1 | 55-64 | 25 | 36 | 25 | 13.30 | 18.65 | 19.53 | 17.16 | 3.37 | 0.269 |
| pUbi:MtDefMd1 | 65-74 | 17 | 11 | 13 | 9.04 | 5.70 | 10.16 | 8.30 | 2.32 | 0.654 |
| pUbi:MtDefMd1 | 75-84 | 8 | 2 | 11 | 4.26 | 1.04 | 8.59 | 4.63 | 3.79 | 0.707 |
| pUbi:MtDefMd1 | 85-94 | 1 | 0 | 6 | 0.53 | 0 | 4.69 | 1.74 | 2.57 | 0.468 |
| pUbi:MtDefMd1 | 95-104 | 0 | 0 | 2 | 0 | 0 | 1.56 | 0.52 | 0.90 | 0.480 |
| pUbi:MtDefMd1 | 105-114 | 0 | 0 | 0 | 0 | 0 | 0 | 0.00 | 0.00 | 0.374 |
| RNAi:MtDefMd1/2 | <15 | 1 | 0 | 0 | 0.55 | 0.00 | 0.00 | 0.18 | 0.32 | 0.374 |
| RNAi:MtDefMd1/2 | 15-24 | 2 | 6 | 0 | 1.09 | 2.24 | 0.00 | 1.11 | 1.12 | 0.349 |
| RNAi:MtDefMd1/2 | 25-34 | 15 | 33 | 12 | 8.20 | 12.31 | 6.94 | 9.15 | 2.81 | 0.122 |
| RNAi:MtDefMd1/2 | 35-44 | 42 | 72 | 28 | 22.95 | 26.87 | 16.18 | 22.00 | 5.40 | 0.554 |
| RNAi:MtDefMd1/2 | 45-54 | 44 | 87 | 50 | 24.04 | 32.46 | 28.90 | 28.47 | 4.23 | 0.083 |
| RNAi:MtDefMd1/2 | 55-64 | 32 | 41 | 42 | 17.49 | 15.30 | 24.28 | 19.02 | 4.68 | 0.186 |
| RNAi:MtDefMd1/2 | 65-74 | 29 | 17 | 18 | 15.85 | 6.34 | 10.40 | 10.86 | 4.77 | 0.899 |
| RNAi:MtDefMd1/2 | 75-84 | 10 | 11 | 8 | 5.46 | 4.10 | 4.62 | 4.73 | 0.69 | 0.611 |
| RNAi:MtDefMd1/2 | 85-94 | 5 | 1 | 15 | 2.73 | 0.37 | 8.67 | 3.93 | 4.28 | 0.940 |
| RNAi:MtDefMd1/2 | 95-104 | 2 | 0 | 0 | 1.09 | 0.00 | 0.00 | 0.36 | 0.63 | 0.231 |
| RNAi:MtDefMd1/2 | 105-114 | 1 | 0 | 0 | 0.55 | 0.00 | 0.00 | 0.18 | 0.32 | 0.626 |
| pPt4:*gusA*int | <15 | 0 | 0 | 0 | 0 | 0 | 0 | 0.00 | 0.00 | - |
| pPt4:*gusA*int | 15-24 | 2 | 4 | 4 | 1.28 | 2.05 | 2.26 | 1.86 | 0.52 | - |
| pPt4:*gusA*int | 25-34 | 31 | 19 | 39 | 19.87 | 9.74 | 22.03 | 17.22 | 6.56 | - |
| pPt4:*gusA*int | 35-44 | 30 | 42 | 68 | 19.23 | 21.54 | 38.42 | 26.40 | 10.48 | - |
| pPt4:*gusA*int | 45-54 | 39 | 34 | 36 | 25.00 | 17.44 | 20.34 | 20.92 | 3.82 | - |
| pPt4:*gusA*int | 55-64 | 13 | 36 | 19 | 8.33 | 18.46 | 10.73 | 12.51 | 5.29 | - |
| pPt4:*gusA*int | 65-74 | 17 | 32 | 6 | 10.90 | 16.41 | 3.39 | 10.23 | 6.54 | - |
| pPt4:*gusA*int | 75-84 | 10 | 17 | 4 | 6.41 | 8.72 | 2.26 | 5.80 | 3.27 | - |
| pPt4:*gusA*int | 85-94 | 10 | 9 | 0 | 6.41 | 4.62 | 0 | 3.68 | 3.31 | - |
| pPt4:*gusA*int | 95-104 | 2 | 2 | 1 | 1.28 | 1.03 | 0.56 | 0.96 | 0.36 | - |
| pPt4:*gusA*int | 105-114 | 2 | 0 | 0 | 1.28 | 0 | 0 | 0.43 | 0.74 | - |

* Two-tailed Student´s t-test
